# Hypoxia-induced sensitisation of TRPA1 in painful dysesthesia evoked by transient hindlimb ischemia/reperfusion in mice

**DOI:** 10.1038/srep23261

**Published:** 2016-03-17

**Authors:** Kanako So, Yuna Tei, Meng Zhao, Takahito Miyake, Haruka Hiyama, Hisashi Shirakawa, Satoshi Imai, Yasuo Mori, Takayuki Nakagawa, Kazuo Matsubara, Shuji Kaneko

**Affiliations:** 1 Department of Molecular Pharmacology, Graduate School of Pharmaceutical Sciences, Kyoto University, 46-29 Yoshida-Shimoadachi-cho, Sakyo-ku, Kyoto 606-8501, Japan; 2 Department of Clinical Pharmacology and Therapeutics, Kyoto University Hospital, 54 Shogoin-Kawahara-cho, Sakyo-ku, Kyoto 606-8507, Japan; 3 Department of Synthetic Chemistry and Biological Chemistry, Graduate School of Enginnering, Kyoto University, Katsura Campus, Nishikyo-ku, Kyoto 615-8510, Japan

## Abstract

Dysesthesia is an unpleasant abnormal sensation, which is often accompanied by peripheral neuropathy or vascular impairment. Here, we examined the roles of transient receptor potential ankyrin 1 (TRPA1) in dysesthesia-like behaviours elicited by transient hindlimb ischemia (15–60 min) by tightly compressing the hindlimb, and reperfusion by releasing the ligature. The paw-withdrawal responses to tactile stimulation were reduced during ischemia and lasted for a while after reperfusion. Hindlimb ischemia/reperfusion elicited spontaneous licking of the ischemic hindpaw that peaked within 10 min. The licking was inhibited by reactive oxygen species (ROS) scavengers, a TRPA1 antagonist, or TRPA1 deficiency, but not by TRPV1 deficiency. In human TRPA1-expressing cells as well as cultured mouse dorsal root ganglion neurons, the H_2_O_2_-evoked TRPA1 response was significantly increased by pretreatment with hypoxia (80 mmHg) for 30 min. This hypoxia-induced TRPA1 sensitisation to H_2_O_2_ was inhibited by overexpressing a catalytically-inactive mutant of prolyl hydroxylase (PHD) 2 or in a TRPA1 proline mutant resistant to PHDs. Consistent with these results, a PHD inhibitor increased H_2_O_2_-evoked nocifensive behaviours through TRPA1 activation. Our results suggest that transient hindlimb ischemia/reperfusion-evoked spontaneous licking, *i.e.* painful dysesthesia, is caused by ROS-evoked activation of TRPA1 sensitised by hypoxia through inhibiting PHD-mediated hydroxylation of a proline residue in TRPA1.

Dysesthesia is an unpleasant abnormal sensation, typically burning, tingling, pricking, and pins-and-needles, which can be spontaneous or provoked by external stimuli. However, dysesthesia is often accompanied by pain, paresthesia (abnormal sensation), and numbness (decrease or loss of sensation)^1^,^2^. Dysesthesia is associated with various diseases, such as diabetic neuropathy, peripheral entrapment neuropathy, polyneuropathy, peripheral arterial disease, and chemotherapy-induced peripheral neuropathy, causing problems during clinical care^3^,^4^,^5^,^6^,^7^. Currently, however, no effective therapeutic drugs are available specifically for treating dysesthesia, mostly because the molecular mechanisms underlying dysesthesia are largely unknown.

Dysesthesia can be induced by either the structural or functional disturbance of primary sensory neurons. In the latter case, the peripheral vascular impairment of peripheral arterial diseases induces cold sensation, dysesthesia, and numbness in the early stage, with increasing pain in the lower extremities as disease progresses^3^,^8^,^9^,^10^. These clinical findings suggest that hypoxia around sensory neurons is responsible for dysesthesia. Thus, several pain models induced by hindlimb ischemia or ischemia/reperfusion have been used. In the chronic post-ischemic pain model, prolonged hindlimb ischemia for 3 h and reperfusion cause mechanical and cold hyperalgesia/allodynia and spontaneous pain behaviours beginning 8 h after the reperfusion and lasting for several weeks, although this model is recognised as a model for complex regional pain syndrome type I^11^,^12^,^13^,^14^. Similarly, ischemic pain models induced by femoral artery thrombus or occlusion have been reported^15^,^16^,^17^. Evidence using these animal models suggests that oxidative stress produced by ischemia or ischemia/reperfusion contributes to the ischemic pain^11^,^17^,^18^,^19^.

Transient receptor potential ankyrin 1 (TRPA1), a nonselective cation channel, is highly expressed in a subset of nociceptive C-fibres and acts as a polymodal nociceptor^20^. TRPA1 is activated by a large number of irritants, reactive oxygen and nitrogen species (ROS/RNS), as well as hyperoxia, through reversible covalent or oxidative modification of cysteine residues at the N-terminal on TRPA1^21^,^22^,^23^,^24^,^25^,^26^,^27^,^28^. By contrast, TRPA1 is also activated by hypoxia; hypoxia inhibits the activity of oxygen-sensitive prolyl hydroxylases (PHDs) and relieves TRPA1 from inhibition by PHD-mediated hydroxylation of a proline residue within the N-terminal ankyrin repeat domain of TRPA1^26^. On the other hand, we previously reported that the acute peripheral neuropathy characteristically induced by oxaliplatin, a platinum-based chemotherapeutic agent, is caused by enhanced responsiveness of TRPA1, but not TRPV1 and TRPM8, in mice^29^.

Recently, Sasaki *et al.* reported that transient hindlimb ischemia in mice induced by compression for 1–10 min and reperfusion provokes spontaneous licking of the ischemic hindpaw and suggests the involvement of ROS generation and TRPA1 activation^30^. However, the mechanisms through which these events occur remain unclear. In the present study, to elucidate the molecular mechanisms underlying behaviours indicative of dysesthesia in mice following transient hindlimb ischemia/reperfusion, we investigated how TRPA1 is activated or sensitised by ROS *in vivo* and *in vitro*. Here, we show that hypoxia induces TRPA1 sensitisation to hydrogen peroxide (H_2_O_2_) by inhibiting the PHD-mediated hydroxylation of an N-terminal proline residue in TRPA1.

## Results

### Hindpaw blood flow is altered during hindlimb ischemia and reperfusion

**Figure 1:**
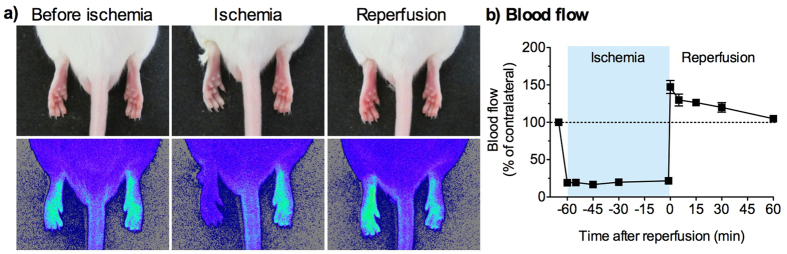
Changes in hindpaw blood flow induced by transient hindlimb ischemia/reperfusion. To induce transient hindlimb ischemia in mice, the left hindlimb just proximal to the ankle joint was compressed by ligation with a string for 60 min. The ligature was rapidly removed for reperfusion. (**a**) Representative photographs (upper panels) and blood flow images (lower panels) of the hindpaws of a mouse before and during ischemia and just after reperfusion. (**b**) The blood flow in the ipsilateral hindpaw was normalised to that in the contralateral hindpaw, and the value obtained just before ischemia served as 100%. Data are presented as means of the percentage ± S.E.M. *n* = 3.

We observed the appearance of the hindpaws and measured hindpaw blood flow in mice during a compressive ligation (ischemia) of the hindlimb with a string and after relief from the compression (reperfusion). During hindlimb ischemia, the ipsilateral hindpaw showed cyanosis, and, immediately after reperfusion, it appeared red and swollen (Fig. 1a). Consistent with these observations, blood flow of the compressed hindpaw during ischemia was reduced to less than 25% of that in the contralateral hindpaw. Immediately after reperfusion, blood flow was transiently increased and gradually returned to the control level within 60 min (Fig. 1b).

### Hypoesthesia during hindlimb ischemia and after reperfusion

**Figure 2:**
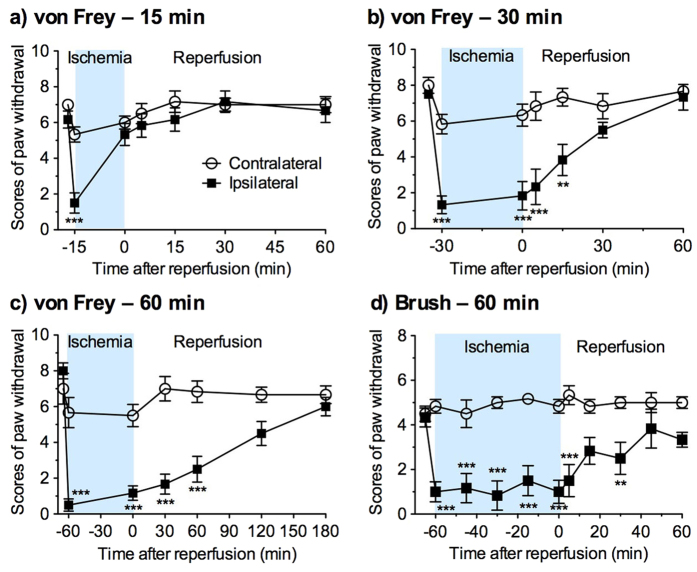
Tactile hypoesthesia during hindlimb ischemia and after reperfusion. (**a–c**) The static tactile responses to a von Frey filament were measured immediately before and during hindlimb ischemia for 15 (**a**), 30 (**b**), and 60 min (**c**), and after reperfusion. (**d**) The dynamic tactile responses to paintbrush stroking were measured immediately before and during hindlimb ischemia for 60 min, and after reperfusion. The paw-withdrawal responses in the contralateral and ipsilateral hindpaws were scored. *n* = 6, **P < 0.01, ***P < 0.001 vs. contralateral.

To determine whether transient hindlimb ischemia and reperfusion affect tactile sensation, the paw-withdrawal responses to von Frey filament and brush stimuli were assessed during ischemia and after reperfusion (Fig. 2). In the von Frey filament test, the scores for the paw-withdrawal response of the contralateral hind paw tended to be decreased only during hindlimb ischemia. The scores for the ipsilateral hind paw were significantly decreased by hindlimb ischemia for 15 min (*F*_1,10_ = 7.24, *p *< 0.05), 30 min (*F*_1,10_ = 20.1, *p *< 0.01), and 60 min (*F*_1,10_ = 37.5, *p *< 0.001). When mice were exposed to hindlimb ischemia for 15 min, the decreased score immediately returned to the control level just after reperfusion. By contrast, when mice were exposed to hindlimb ischemia for 30 and 60 min, the scores of the paw-withdrawal responses were significantly reduced until 15 and 60 min after the reperfusion, respectively, compared with those for the contralateral hind paw, and then gradually returned to control levels. Similarly, in the brush test, hindlimb ischemia/reperfusion for 60 min significantly reduced the scores of the paw-withdrawal responses (*F*_1,10_ = 76.5, *p *< 0.001). The significant decreases were observed until 30 min after reperfusion.

### Hindlimb ischemia/reperfusion evokes spontaneous licking behaviour

**Figure 3:**
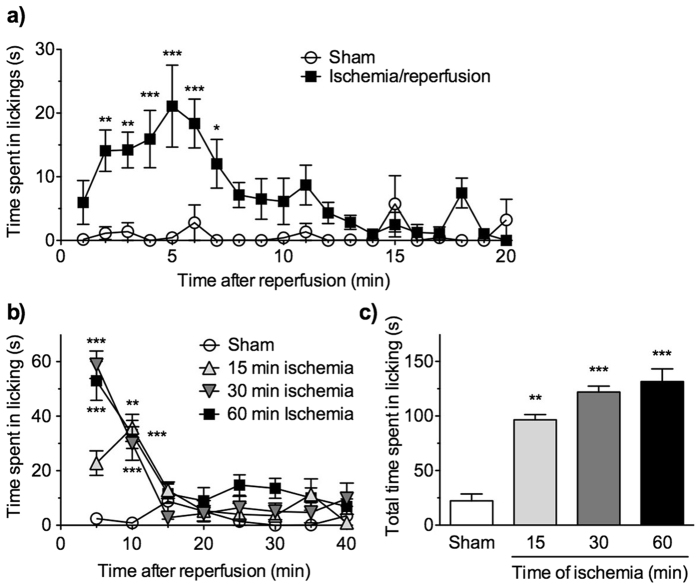
Spontaneous licking following transient hindlimb ischemia/reperfusion. Time course of spontaneous licking after reperfusion following a transient hindlimb ischemia for 15, 30 and 60 min. (**a**) The time spent licking the ipsilateral hindpaw was measured every 1 min for 20 min after reperfusion following 60-min hindlimb ischemia (*n* = 7) or sham treatment (*n* = 6). *P < 0.05, **P < 0.01, ***P < 0.001 vs sham treatment. (**b,c**) The time spent licking the ipsilateral hindpaw every 5 min for 40 min (**b**) and total time spent licking for 40 min (**c**) was measured after reperfusion following 15, 30 or 60-min hindlimb ischemia/reperfusion or sham treatment. *n* = 4–15, **P < 0.01, ***P < 0.001 vs sham treatment.

When mice were exposed to hindlimb ischemia for 60 min followed by relief from the ischemia, spontaneous licking of the ipsilateral hindpaw was observed immediately after reperfusion, whereas other escape behaviours, such as flicking, were rarely observed (Supplementary Video). When recorded every 1 min for 20 min, the duration of licking was significantly increased (*F*_1,11_ = 95.4, *p *< 0.001). The significant increases were observed during 2–7 min after reperfusion, compared with that in sham-treated mice, and the licking peaked at 5 min (Fig. 3a). When mice were exposed to hindlimb ischemia for 15, 30 or 60 min and the time spent licking was recorded every 5 min for 40 min after reperfusion, the duration of licking was significantly increased (*F*_3,29_ = 58.6, *p *< 0.001) at 5 and 10 min after reperfusion, compared with that in sham-treated mice. The licking once disappeared at 15 and 20 min, but was slightly increased again thereafter until 40 min after the reperfusion following 60-min hindlimb ischemia. However, such biphasic licking was not observed by 15- and 30-min hindlimb ischemia (Fig. 3b). The total duration of the time spent licking during 40 min was significantly increased at an ischemic time-dependent manner (Fig. 3c; *F*_3,29_ = 21.4, *p *< 0.001). When analysed in the early (0–10 min) and delayed phases (10–40 min) of licking, the former was significantly increased by either 15-, 30- or 60-min hindlimb ischemia, while the latter was significantly increased only by 60-min, but not 15- and 30-min, hindlimb ischemia (Supplementary Fig. S1).

We selected a hindlimb ischemia of 60 min as our model of dysesthesia accompanied by tactile hypoesthesia and the delayed phase of spontaneous licking for the following experiments.

### Involvement of ROS and TRPA1 in hindlimb ischemia/reperfusion-evoked licking

**Figure 4:**
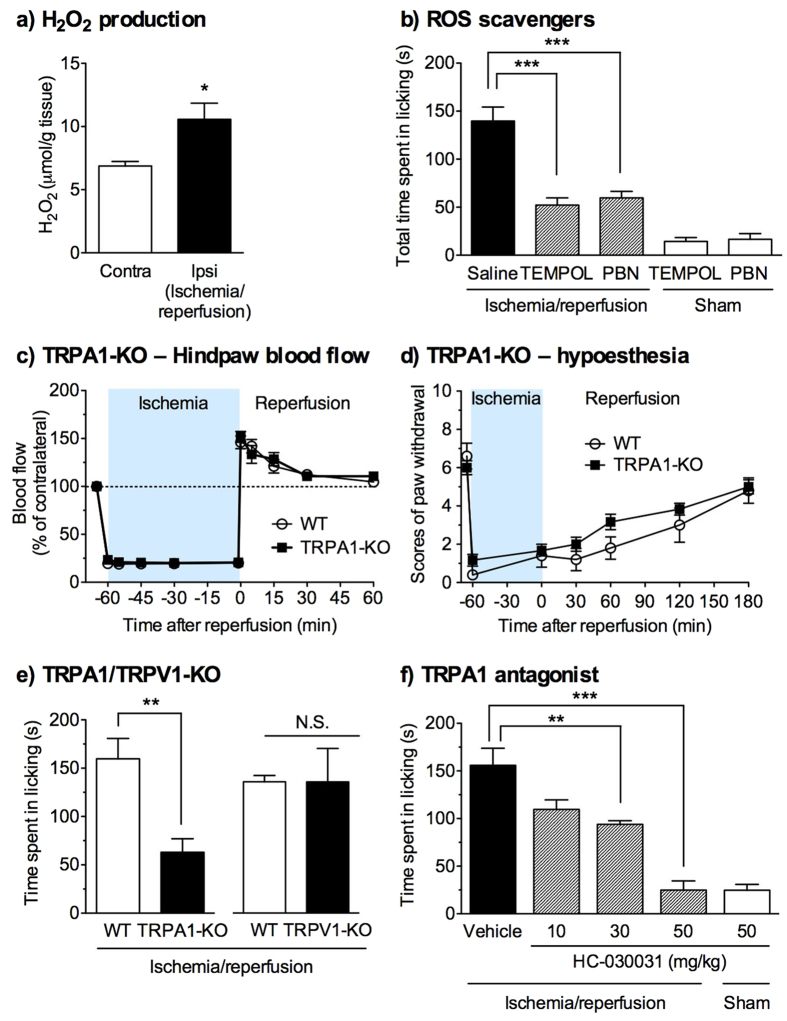
Involvement of reactive oxygen species (ROS) and TRPA1 in the hindlimb ischemia/reperfusion-evoked spontaneous licking. (**a**) H_2_O_2_ production following the hindlimb ischemia/reperfusion. The H_2_O_2_ contents (nmol/mg protein) in the contralateral (contra) and ipsilateral (ipsi) paws 5 min after reperfusion following 60-min hindlimb ischemia were measured. *n* = 5, *P < 0.05, vs contralateral. (**b**) Effects of ROS scavengers on the hindlimb ischemia/reperfusion-evoked spontaneous licking. 4-hydroxy-2,2,6,6-tetramethyl-1-piperidinyloxy (TEMPOL; 250 mg/kg), α-phenyl-*tert*-butyl nitrone (PBN; 100 mg/kg), or vehicle was administered intraperitoneally (i.p.) 15 min before reperfusion following 60-min hindlimb ischemia or sham treatment. The time spent licking was measured for 40 min after reperfusion following hindlimb ischemia of 60 min. *n* = 6, ***P < 0.001. (**c**) Wild-type (WT) or TRPA1-knockout (KO) mice were subjected to hindlimb ischemia/reperfusion, and blood flow was measured in both the contralateral and ipsilateral hindpaws. Blood flow in the ipsilateral hindpaw was normalised to that in the contralateral hindpaw, and the value obtained just before ischemia served as 100%. *n* = 5. No difference in blood flow is observed between WT and TRPA1-KO mice. (**d**) The static tactile responses to a von Frey filament in WT and TRPA1- KO mice were measured during hindlimb ischemia and after reperfusion. The paw-withdrawal responses in the ipsilateral hindpaw were scored. *n* = 5–6. (**e**) The time spent licking in WT, TRPA1-KO, or TRPV1-KO mice was measured for 40 min following hindlimb ischemia/reperfusion. *n* = 5–9. **P < 0.01. N.S. = not significant. (**f**) The TRPA1 antagonist HC-030031 (10, 30, and 50 mg/kg) or vehicle was administered i.p. immediately before initiating ischemia or in sham-treated mice. The time spent licking was measured for 40 min following hindlimb ischemia/reperfusion. *n* = 5–7, **P < 0.01, ***P < 0.001.

Following ischemia, reperfusion of oxygenated blood into ischemic tissues induces ROS production, which causes oxidative injury^31^,^32^. In the hindlimb ischemic/reperfused paws, the content of H_2_O_2_ was significantly increased compared with that in the contralateral paws (Fig. 4a). To determine whether ROS is involved in the transient hindlimb ischemia/reperfusion-evoked licking, we examined the effects of the membrane-permeable ROS scavengers 4-hydroxy-2,2,6,6-tetramethyl-1-piperidinyloxy (TEMPOL) and α-phenyl-*tert*-butyl nitrone (PBN). Compared with the administration of saline, the intraperitoneal (i.p.) administration of TEMPOL (250 mg/kg) or PBN (100 mg/kg) 15 min before reperfusion significantly attenuated the duration of licking in ischemic/reperfused mice (*F*_2,15_ = 22.7, *p *< 0.001), with no effect in sham-treated mice (Fig. 4b). When analysed in the early (0–10 min) and delayed phases (10–40 min) of licking, both phases were significantly attenuated by TEMPOL or PBN (Supplementary Fig. S2). By contrast, neither TEMPOL (250 mg/kg) nor PBN (100 mg/kg) affected the nocifensive behaviour evoked by an intraplantar injection of a TRPA1 agonist, allyl isothiocyanate (AITC) (Supplementary Fig. S3). These findings suggest that TEMPOL and PBN can attenuate the licking behaviour via ROS production by hindlimb ischemia/reperfusion, but not TRPA1 stimulation-evoked nocifensive behaviours.

Next, we investigated the roles of redox-sensitive TRPA1^26^,^27^ in hindlimb ischemia/reperfusion-evoked licking by using TRPA1-knockout (TRPA1-KO) mice. There was no significant difference in the alterations of the hindpaw blood flow between wild-type (WT) and TRPA1-KO mice (*F*_1,8_ = 0.16, *p* = 0.70) (Fig. 4c). The paw-withdrawal response to von Frey filament stimulation in WT and TRPA1-KO mice were similarly reduced during hindlimb ischemia and after reperfusion. There was no significant difference between them (*F*_1,9_ = 1.78, *p* = 0.21) (Fig. 4d). By contrast, the duration of the hindlimb ischemia/reperfusion-evoked licking was significantly attenuated in TRPA1-KO mice compared with that in WT mice, whereas it remained unchanged in TRPV1-KO mice (Fig. 4e). The i.p. administration of HC-030031, a TRPA1 antagonist (10, 30 and 50 mg/kg), immediately before initiating ischemia significantly attenuated the duration of licking in a dose-dependent manner (*F*_3,21_ = 20.0, *p *< 0.011). Compared with the vehicle-administered group, significant effects of HC-030031 were observed at 30 and 50 mg/kg, and no effect of HC-030031 was observed in sham-treated mice (Fig. 4f). When analysed in the early (0–10 min) and delayed phases (10–40 min) of licking, both phases were significantly attenuated by TRPA1 deficiency or HC-030031, but not by TRPV1 deficiency (Supplementary Fig. S4).

### Hypoxia enhances H_2_O_2_-evoked TRPA1 activation

**Figure 5:**
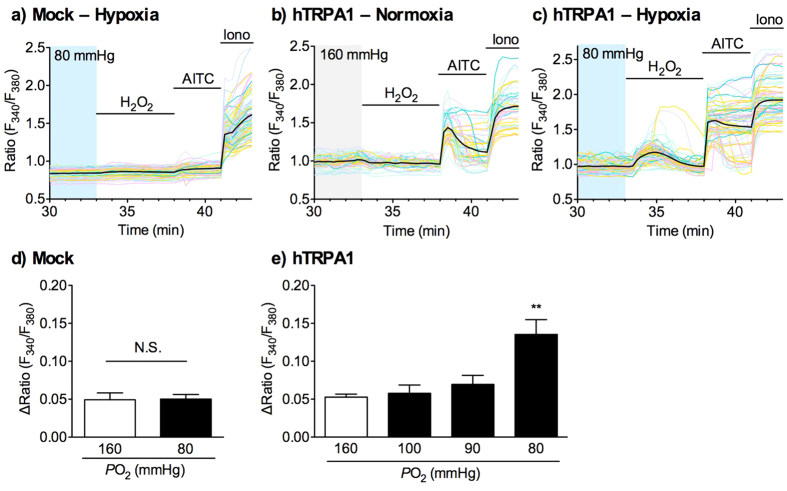
Effects of hypoxia on H_2_O_2_-evoked TRPA1 activation in hTRPA1-expressing cells. HEK293 cells transfected with vector (**a**,**d**; mock) or hTRPA1 cDNA (**b**,**c**,**e**) were pretreated with normoxia (160 mmHg) or hypoxia (100, 90, or 80 mmHg) for 30 min, and then exposed to H_2_O_2_ (10 μM) for 5 min, as shown in Supplementary Fig. S5. (**a–c**) Representative images of [Ca^2+^]_i_ changes are shown as F_340_/F_380_ ratio. F_340_/F_380_ ratio in each cell is shown as pale-coloured lines, and the average of F_340_/F_380_ ratio is shown as a black solid line. (**d,e**) Statistical analyses of H_2_O_2_-evoked [Ca^2+^]_i_ increases were calculated as the average of the maximal ΔRatio during the 5 min H_2_O_2_ application period (33–38 min) obtained from 5–6 independent experiments, as described in Materials and Methods. AITC, allyl isothiocyanate, 100 μM; Iono, ionomycin, 3 μM. **P < 0.01, compared with normoxia (160 mmHg). N.S. = not significant.

Using fluorometric Ca^2+^ imaging analysis, we investigated the effects of hypoxia on TRPA1 activation in response to H_2_O_2_ in HEK293 cells expressing human TRPA1 (hTRPA1). When the oxygen (O_2_) concentration in Krebs-Ringer buffer was abruptly decreased from a partial O_2_ pressure (*P*O_2_) of 160 mmHg (21% O_2_) to 80 mmHg (10.5% O_2_), the intracellular Ca^2+^ concentration ([Ca^2+^]_i_) was rapidly increased in many hTRPA1-expressing cells (Supplementary Fig. S5), consistent with a previous report^26^. However, when the O_2_ concentration in the buffer was gradually decreased to a *P*O_2_ of 80 mmHg, only a few hTRPA1-expressing cells responded (Supplementary Fig. S6). The O_2_ concentration of atmospheric *P*O_2_ (160 mmHg) was decreased over 20–30 min to the hypoxic *P*O_2_ of 80 mmHg. Those cells responding to hypoxia were excluded from the analyses. Following pretreatment with hypoxia at a *P*O_2_ of 80–100 mmHg for 30 min, we rapidly returned the O_2_ concentration to a *P*O_2_ of approximately 160 mmHg and concomitantly applied H_2_O_2_ (10 μM) dissolved in normoxic buffer. In order to better detect alterations of TRPA1 function, we set a low H_2_O_2_ concentration of 10 μM. In mock-transfected cells, the application of H_2_O_2_ had no effect on [Ca^2+^]_i_ following pretreatment with either normoxia (160 mmHg) or hypoxia (80 mmHg) (Fig. 5a,d). In hTRPA1-expressing cells pretreated with normoxia (160 mmHg) for 30 min, the application of 10 μM H_2_O_2_ had no or little effect on [Ca^2+^]_i_ (Fig. 5b). By contrast, robust H_2_O_2_-evoked [Ca^2+^]_i_ increases were observed in many cells pretreated with hypoxia (80 mmHg) for 30 min (Fig. 5c). Quantitative analyses revealed that pretreating with hypoxia at a *P*O_2_ of 100 mmHg (13.2% O_2_), 90 mmHg (11.8% O_2_), or 80 mmHg (10.5% O_2_) for 30 min significantly enhanced the change in the F_340_/F_380_ ratio (ΔRatio) of the H_2_O_2_-evoked [Ca^2+^]_i_ increase in an O_2_ concentration-dependent manner (*F*_3,20_ = 9.02, *p *< 0.001). A significant difference was observed at a *P*O_2_ of 80 mmHg (Fig. 5e).

**Figure 6:**
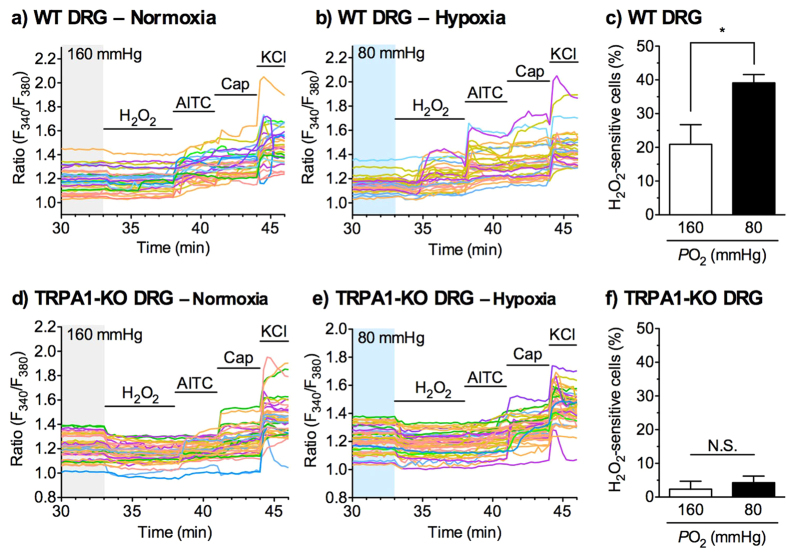
Effects of hypoxia on the H_2_O_2_-evoked TRPA1 activation in cultured mouse dorsal root ganglion (DRG) neurons. Cultured mouse DRG neurons prepared from WT (**a–c**) or TRPA1-KO (**d–f**) mice were pretreated with normoxia (160 mmHg) or hypoxia (80 mmHg) for 30 min, and then exposed to H_2_O_2_ (100 μM) for 5 min, as shown in Supplementary Fig. S5. (**a,b,d,e**) Representative images of [Ca^2+^]_i_ changes are shown as F_340_/F_380_ ratio. (**c,f**) Statistical analyses of H_2_O_2_-evoked [Ca^2+^]_i_ increases were calculated as the percentage of H_2_O_2_-sensitive cells in 50 mM KCl-sensitive neurons obtained from 5–6 independent experiments, described in the Materials and Methods. AITC, allyl isothiocyanate, 100 μM; Cap, capsaicin, 0.3 μM; KCl, 50 mM. *P < 0.05. N.S. = not significant.

Similarly, the effects of hypoxia pretreatment on the H_2_O_2_-evoked Ca^2+^ responses were investigated in cultured dorsal root ganglion (DRG) neurons isolated from WT or TRPA1-KO mice. In WT DRG neurons pretreated with normoxia (160 mmHg) for 30 min, the application of 100 μM H_2_O_2_ increased [Ca^2+^]_i_ in 20.9 ± 5.8% of the DRG neurons. Pretreatment with hypoxia (80 mmHg) for 30 min enhanced the H_2_O_2_-evoked [Ca^2+^]_i_ increase. The number of H_2_O_2_-sensitive neurons pretreated with hypoxia was significantly greater than that pretreated with normoxia (Fig. 6a–c). By contrast, few TRPA1-KO DRG neurons responded to 100 μM H_2_O_2_. Pretreatment with hypoxia (80 mmHg) for 30 min did not affect the number of H_2_O_2_-sensitive cells. The number of H_2_O_2_-sensitive cells in TRPA1-KO DRG neurons did not differ under normoxic and hypoxic conditions (Fig. 6d–f).

### Involvement of PHD and an N-terminal proline residue of TRPA1 in hypoxia-induced TRPA1 sensitisation to H_2_O_2_

**Figure 7:**
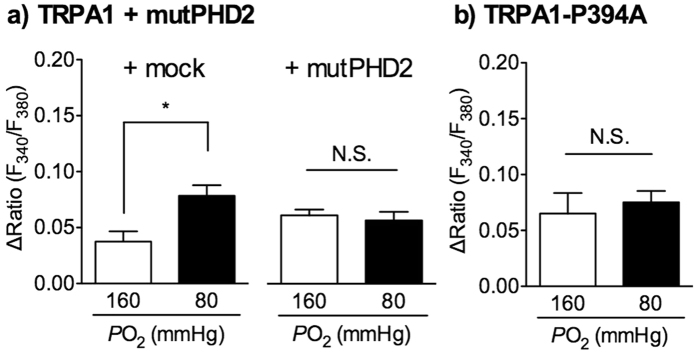
Involvement of prolyl hydroxylase (PHD) and Pro^394^ in the hypoxia-induced TRPA1 sensitisation to H_2_O_2_. (**a,b**) HEK293 cells cotransfected with human TRPA1 (hTRPA1) and a vector (mock) or a dominant negative mutant of hPHD2 (mutPHD2) cDNAs (**a**), or transfected with hTRPA1-P394A cDNA (**b**), were pretreated with normoxia (160 mmHg) or hypoxia (80 mmHg) for 30 min, and then exposed to H_2_O_2_ (10 μM). Statistical analyses of H_2_O_2_-evoked [Ca^2+^]_i_ increases were calculated as the average of the ΔRatio during the 5 min H_2_O_2_ application obtained from five independent experiments. *P < 0.05. N.S.  = not significant.

Hypoxia diminishes the activity of PHDs^33^,^34^ and activates TRPA1 through relief from PHD-mediated hydroxylation of proline residue 394 (Pro^394^) of hTRPA1^26^. To determine whether the hypoxia-enhanced TRPA1 response to H_2_O_2_ is mediated through the PHD-inhibition and dehydroxylation of the hTRPA1 Pro^394^, we used a catalytically inactive dominant negative mutant of human PHD2 (mutPHD2) and an hTRPA1 Pro^394^ mutant to alanine (hTRPA1-P394A), which has no interaction with PHDs^26^. In HEK293 cells cotransfected with hTRPA1 and a vector (mock), pretreatment with hypoxia (80 mmHg) for 30 min significantly enhanced the H_2_O_2_-evoked [Ca^2+^]_i_ increase compared with that observed under normoxic conditions (160 mmHg). In cells cotransfected with hTRPA1 and mutPHD2, the H_2_O_2_-evoked [Ca^2+^]_i_ increase tended to be high, even following normoxia pretreatment. However, hypoxia pretreatment did not affect the H_2_O_2_-evoked [Ca^2+^]_i_ increase, and the response of the normoxia- and hypoxia-pretreated cells was not different (Fig. 7a). Similarly, in hTRPA1-P394A-expressing cells, the H_2_O_2_-evoked [Ca^2+^]_i_ increase tended to be high, even following normoxia pretreatment, but there was no difference in the response between normoxia- and hypoxia-pretreated cells (Fig. 7b).

### PHD inhibitor enhances H_2_O_2_-evoked nocifensive behaviours through TRPA1 activation

**Figure 8:**
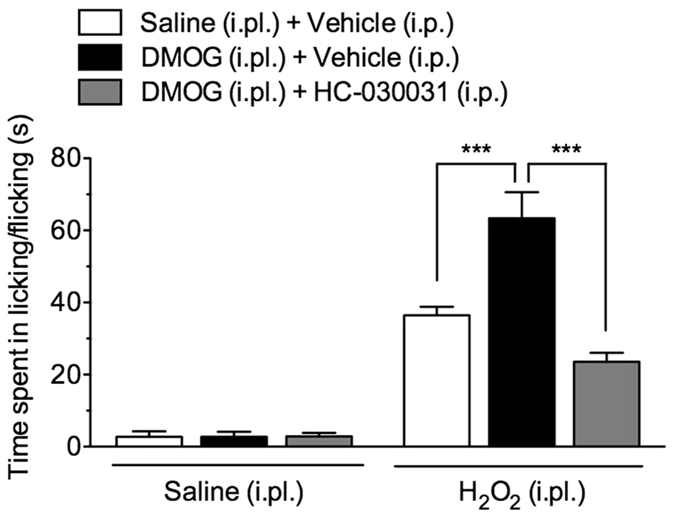
Effect of a prolyl hydroxylase (PHD) inhibitor on H_2_O_2_-evoked nocifensive behaviours. Mice were injected i.pl. with the PHD inhibitor dimethyloxalylglycine (DMOG; 25 μg/paw) or saline. One hour after the pretreatment, mice were injected with H_2_O_2_ (1.5%, 20 μL/paw) or saline. The time mice spent in H_2_O_2_-evoked nocifensive behaviours, such as ipsilateral paw licking and flicking, was measured for 5 min. The TRPA1 antagonist HC-030031 (100 mg/kg) or vehicle was administered i.p. 30 min before the H_2_O_2_ injection. *n* = 5–8, ***P < 0.001.

Finally, we investigated whether the PHD inhibitor dimethyloxalylglycine (DMOG) enhances TRPA1-mediated nocifensive behaviours in mice. In mice pretreated intraplantarly (i.pl.) with vehicle, an i.pl. injection of H_2_O_2_ (0.5%, 20 μL/paw), but not saline, evoked nocifensive behaviours, including licking and flicking of the injected paw. The time spent in H_2_O_2_-evoked nocifensive behaviour was significantly increased in mice pretreated with DMOG (25 μg/paw) compared with that in vehicle-pretreated mice. Pre-administration of HC-030031 (100 mg/kg, i.p.) significantly attenuated the increased DMOG-induced time spent in H_2_O_2_-evoked nocifensive behaviour but had no effect in vehicle-pretreated mice (Fig. 8).

## Discussion

In humans, reperfusion following ischemia of an upper or lower limb often induces dysesthesia accompanied by pain and numbness. In this study, we provided the first evidence that peripheral ischemia/reperfusion-evoked spontaneous licking, *i.e.,* painful dysesthesia observed in mice, is caused by the hypoxia-induced TRPA1 sensitisation to ROS through inhibition of PHD-mediated hydroxylation of an N-terminal proline residue in TRPA1.

A variety of animal models of semi-permanent hindlimb ischemia are widely used for evaluation of the pathophysiology of peripheral arterial disease^35^,^36^,^37^,^38^ as well as of ischemic pain^15^,^16^. In addition, a body of evidence suggests that reperfusion following transient hindlimb ischemia for several hours can induce long-lasting post-ischemic pain, although the onset and peak time are delayed until several days after the reperfusion^11^,^12^,^17^,^19^. By contrast, the present results demonstrated that reperfusion following transient hindlimb ischemia for 15–60 min rapidly evoked spontaneous licking, which then immediately ceased. Considering its rapid onset and offset, the ischemia/reperfusion-evoked spontaneous licking is different from the delayed onset of long-lasting post-ischemic pain. The spontaneous liking may be caused by the functional neuropathy, rather than structural injury, induced by transient hindlimb ischemia/reperfusion. Consistently, Sasaki *et al.* reported that shorter hindlimb ischemia (1–10 min) evokes similar spontaneous licking, which appeared during the first 10-min period immediately after the reperfusion, but then disappeared^30^. By contrast, we found that longer hindlimb ischemia (60 min) evoked biphasic spontaneous licking, which was composed of early (0–10 min) and delayed phases (10–40 min). The former is similar to the previous report^30^, while the latter is likely to be elicited only by longer hindlimb ischemia. Another characteristic feature of the present model is that the tactile hypoesthesia lasted for a while, even after reperfusion following longer hindlimb ischemia of more than 30 min, indicating that spontaneous licking accompanies hypoesthesia. Although it may appear that these sensations are conflicting, clinical findings clearly show that dysesthesia includes or accompanies various sensations, such as paresthesia and numbness^1^,^2^. However, the duration of hypoesthesia after reperfusion is dependent on the time of compressive ischemia, but independent of the time of ischemia/reperfusion-evoked spontaneous licking. Furthermore, TRPA1 deficiency reduced ischemia/reperfusion-evoked licking, but not hypoesthesia. Taken together, our data suggest that dysesthesia and hypoesthesia are independent and are based on different mechanisms.

Accumulating evidence suggests that ROS produced by ischemia/reperfusion is a key player in post-ischemic pain^11^,^17^,^18^. Ischemia/reperfusion-induced injury of sensory nerves is caused by excess ROS, which can lead to lipid peroxidation, protein oxidization, calcium overload, DNA damage, and the production of inflammatory cytokines^32^,^39^. In addition to these ROS-mediated cytotoxic effects, ROS can specifically activate redox-sensitive TRP channels, including TRPA1 and TRPV1^27^. The present findings suggest that ROS produced by transient hindlimb ischemia/reperfusion evokes spontaneous licking through activation of TRPA1, but not TRPV1. This finding is supported by a previous finding that mitochondrial dysfunction preferentially activates TRPA1, but not TRPV1, through ROS production derived from mitochondria^40^. The ROS/TRPA1-mediated spontaneous licking behaviour predominantly represents the painful aspect of dysesthesia. We found that both early and delayed phases of spontaneous licking were suppressed by ROS scavengers and TRPA1 blockade. The former is likely to be elicited by ROS and TRPA1 activation, similar to the previous report^30^. However, the latter mechanism is still unclear, because it may be due to secondary responses induced by early ROS/TRPA1 activation, like the second phase of formalin-induced nocifensive behaviors. On the other hand, TRPA1 has been shown to be involved in the vasoconstriction and vasodilatation responses to cold exposure via ROS production and calcitonin gene-related peptide/substance P release, respectively^41^. The present results suggest, however, that TRPA1 is unlikely to be involved in the decrease, transient increase, and recovery of hindpaw blood flow induced by hindlimb compressive ligation and its relief.

The major finding of this study is that hypoxia enhances the sensitivity of TRPA1 to ROS. To mimic the transient hindlimb ischemia/reperfusion *in vitro*, TRPA1-expressing cells or mouse cultured DRG neurons were pretreated with hypoxia and then subjected to H_2_O_2_ with reoxygenation. TRPA1 is activated by H_2_O_2_, as well as hyperoxia, through oxidative modification of the cysteine residues at the N-terminal of TRPA1^26^,^27^,^28^,^42^,^43^, whereas hypoxia relieves TRPA1 from inhibition by PHD-mediated hydroxylation, leading to TRPA1 activation^26^. However, we found that pretreatment with hypoxia enhanced H_2_O_2_-evoked TRPA1 activation, *i.e.*, TRPA1 sensitisation to ROS. The *P*O_2_ that induced TRPA1 sensitisation was approximately 80 mmHg, which corresponds to that for hypoxia-induced TRPA1 activation^26^, suggesting that hypoxia-induced TRPA1 activation and sensitisation are closely related. In TRPA1-KO DRG neurons, the H_2_O_2_-evoked [Ca^2+^]_i_ response was not observed, even after pretreatment with hypoxia. This result suggests that hypoxia selectively enhances the sensitivity of TRPA1, but not other ROS-sensitive channels, in DRG neurons^42^,^43^. Thus, TRPA1 plays a pivotal role in ROS-mediated responses of sensory neurons induced by peripheral ischemia/reperfusion. However, we can not fully exclude the possible involvement of other mechanisms than ROS-mediated TRPA1 activation in the ischemia/reperfusion-evoked spontaneous licking, because TRPA1 deficiency did not completely vanish the behaviour.

The TRPA1 Pro^394^ residue is normally hydroxylated by PHDs in normoxia, which induces internalization of TRPA1 and suppresses TRPA1 activation. When PHD activity is inhibited by hypoxia, unmodified TRPA1 proteins are inserted into the plasma membrane and then activated^26^. In the present study, we found that the hypoxia-induced TRPA1 sensitisation to ROS was attenuated by overexpression of mutPHD2 or in hTRPA1-P394A-expressing cells. These results suggest that the hypoxia-induced TRPA1 sensitisation is caused by inhibiting the PHD-mediated hydroxylation of the TRPA1 Pro^394^ residue, similar to the mechanism of hypoxia-induced TRPA1 activation. This finding is further supported by our *in vivo* behavioural experiments in which H_2_O_2_-evoked nocifensive behaviour through TRPA1 was enhanced by a PHD inhibitor. However, further investigations will be needed to determine whether inhibiting the hydroxylation of Pro^394^ increases TRPA1 localization in the plasma membrane or enhances the ROS-mediated oxidative sensitivity of N-terminal cysteine residues on TRPA1.

In conclusion, our data suggest that post-ischemic painful dysesthesia following a transient compressive hindlimb ischemia/reperfusion is caused by ROS-evoked activation of TRPA1 sensitised by hypoxia through inhibition of PHD-mediated hydroxylation of the TRPA1 Pro^394^ residue. The present findings elucidate a molecular mechanism underlying peripheral vascular neuropathy and suggest that hypoxia-induced TRPA1 sensitisation plays a pivotal role in painful dysesthesia.

## Materials and Methods

### Animals

All experiments were performed according to the ethical guidelines recommended by the Kyoto University Animal Research Committee. The protocol was approved by the Kyoto University Animal Research Committee (permit number: 2014–45, 2015–38). All efforts were made to minimise the number of animals used and limit experimentation to only what was necessary. Male ICR mice aged 6–7 weeks were purchased from Japan SLC (Shizuoka, Japan). TRPA1-KO mice were obtained from Jackson Laboratory (Bar Harbor, ME, USA) and genotyped as previously described^23^. TRPV1-KO mice were a gift from Dr. David Julius (University of California, San Francisco, USA). Each KO mouse line was backcrossed with C57BL/6J mice for 7 to 10 generations to eliminate any background effects on the phenotypes. All mice were housed under constant ambient temperature (24 ± 1 °C) and humidity (55 ± 10%), with alternate light–dark cycles from 8:00 a.m. to 8:00 p.m. Food and water were freely available.

### Drugs and reagents

TEMPOL and PBN (Sigma-Aldrich, St. Louis, MO, USA) were freshly dissolved in sterile saline. HC-030031 (Haoyuan Chemexpress, Shanghai, China) was freshly suspended in 0.5% methylcellulose (Wako Pure Chemical Industries, Osaka, Japan). DMOG (Frontier Scientific, Logan, UT, USA) and H_2_O_2_ (Wako) were freshly dissolved in sterile saline for intraplantar administration. Allyl isothiocyanate (AITC; Wako) and capsaicin (Sigma-Aldrich) were dissolved in dimethyl sulfoxide in Krebs-Ringer buffer for Ca^2+^ imaging experiments. All other reagents were of the best available quality from commercial sources.

### Dysesthesia model induced by transient hindlimb ischemia/reperfusion

Hindlimb ischemia was induced by compression with a string. Under isoflurane anaesthesia, the mouse’s left hindlimb just proximal to the ankle joint was tightly ligated using a string (No. 20; Pocket, Sanda, Japan) to induce ischemia (Fig. 1a). The mice were returned to an observation cylinder to recover from the anaesthesia, except for the experiments measuring hindpaw blood flow. To keep the mice from biting and cutting the string, they were fitted with an Elizabethan collar (Lomir Biomedical Inc., Malone, NY) during hindlimb compression. After 15–60 min of ischemia, the ligature was quickly removed by cutting the string to allow for hindlimb reperfusion. Sham-treated mice received identical treatment, except their hindlimbs were not compressed with a string.

### Measurement of hindpaw blood flow

Mice were anaesthetised with pentobarbital (64.8 mg/kg, i.p.). The blood flow of non-ischemic right (contralateral) and ischemic left (ipsilateral) hindpaws was measured using a laser speckle blood flow analysing system (OMEGA ZONE, Omega wave Co., Tokyo, Japan) at the times indicated. To avoid data variations caused by ambient light and temperature, the blood flow in the ipsilateral hindpaw was normalised to that in the contralateral hindpaw.

### Behavioural tests

#### Spontaneous licking induced by a transient hindlimb ischemia/reperfusion

Mice were individually habituated to the experimental conditions in an acrylic observation cylinder (diameter, 30 cm; height, 50 cm) for approximately 1 h before reperfusion. The mice receiving transient hindlimb ischemia/reperfusion or sham treatment were individually placed in the centre of the observation cylinders, and their spontaneous behaviours were observed. The time spent licking the ipsilateral hindpaw was recorded every 1 min for 20 min or 5 min for 60 min, and the total time spent licking was calculated for 40 min after the reperfusion.

#### Tactile sensitivities to static and dynamic stimuli

Mice were individually habituated to the experimental conditions of a metal mesh floor in a small cylinder (diameter, 7.5 cm; height, 10 cm) for at least 30 min. Their static tactile sensitivity was assessed for a few seconds using a 1 g von Frey filament (Stoelting Co., Wood Dale, IL, USA) applied until it bent slightly on the middle plantar surface of each hindpaw. The dynamic tactile sensitivity was assessed by lightly stroking the dorsal surface of each hindpaw with a paintbrush (Φ, 10 mm; Pentel, Tokyo, Japan). The paw-withdrawal response was graded using the following scores: 0, no response; 1, moderate effort to avoid the probe, such as licking the stimulated paw; and 2, vigorous effort to escape the stimulus, such as jumping, shaking the hindpaw, or biting at the probe or the stimulated paw. One trial involved five applications of a von Frey filament or a paintbrush every 3 or 4 s, each of which was scored as 0, 1, or 2. The trial was evaluated based on a total score of 0 to 10 at culmination.

#### Nocifensive behaviour evoked by intraplantar injection of H_2_O_2_

Mice were individually habituated to the experimental conditions in an acrylic observation cylinder for approximately 1 h before H_2_O_2_ injection. Either H_2_O_2_ (1.5%, 20 μL/paw) or saline (20 μL/paw) was subcutaneously injected into the plantar surface of the right hindpaw, and the time spent in nocifensive behaviours, such as licking and flicking of the injected hindpaw, was measured for 5 min. DMOG (25 μg/paw) or saline (20 μL/paw) was subcutaneously injected into the plantar surface of the hindpaw 60 min before the H_2_O_2_ injection. HC-0300311 (100 mg/kg) or vehicle (0.5% methylcellulose) was administered i.p. 30 min before the H_2_O_2_ injection.

### Measurement of H_2_O_2_ content

The content of H_2_O_2_ in the ischemic/reperfused paws was measured, as previously described^44^ with sight modifications. Mice were exposed to hindlimb ischemia for 60 min, and sacrificed by cervical dislocation 5 min after reperfusion. The plantar tissues were immediately removed from the contralateral and ipsilateral mouse hindpaws, frozen in liquid nitrogen, and stored at −80 °C until use. They were homogenized with a Polytron homogenizer in ice-cold 50 mM phosphate buffer (pH 7.4) with 0.5% hexadecyltrimethylammonium bromide. The homogenates were centrifuged at 15,000 rpm, 4 °C for 2 min to remove debris. The supernatants were filtered through an Amicon Ultra Centrifugal Filter Unit with an Ultracel-100 membrane (Merck Millipore, Billerica, MA) by centrifugation at 15,000 rpm, 4 °C for 2 min. The H_2_O_2_ content in the flow-through was determined with an Amplex Red Assay Kit (Invitrogen, Life Technologies, Carlsbad, CA), in accordance with the manufacturer’s protocol.

### HEK293 cell culture and cDNA transfection

HEK293 cells were cultured in Dulbecco’s modified Eagle’s medium (DMEM; Sigma-Aldrich) supplemented with 10% heat-inactivated foetal bovine serum (Sigma-Aldrich), and maintained at 37 °C in a humidified incubator set at 5% CO_2_. Recombinant plasmids for hTRPA1, hTRPA1-P394A and mutPHD2 in pCI-neo mammalian expression vectors (Promega Co., Madison, WI) were constructed as previously described^26^. HEK293 cells were transfected with pCI-neo-hTRPA1 (2.0 μg) or pCI-neo-hTRPA1-P394A (2.0 μg) with pEGFP-C3 (0.5 μg), or cotransfected with pCI-neo-hTRPA1 (1.12 μg), pCI-neo-mutPHD2 (1.12 μg) and pEGFP-C3 (0.28 μg) by using Lipofectamine 2000 (Life Technologies, Carlsbad, CA). Two days after the transfection, cells were removed from the culture dish and plated onto coverslips coated with poly-L-lysine.

### Primary cultures of mouse DRG neurons

Primary cultures of mouse DRG neurons were prepared as previously described^29^. C57BL/6 J (WT) or TRPA1-KO mice (aged 6–8 weeks) were decapitated, and bilateral L1–L6 DRG were immediately harvested. The DRG were incubated for 1 h at 37 °C in Hank’s balanced salt solution containing (in mM) 137 NaCl, 5.4 KCl, 0.34 Na_2_HPO_4_, 0.44 KH_2_PO_4_, 5.6 D-glucose, and 2.4 HEPES (adjusted to pH 7.4 with NaOH) as well as 0.3% collagenase and 0.4% dispase. A Percoll (Sigma-Aldrich) gradient was used to separate DRG neurons from myelin and nerve debris as follows. Solutions of 30% and 60% Percoll were prepared with L15 medium. The 30% Percoll was gently layered over the 60% Percoll solution, and the cell suspension was layered over the Percoll gradient. After 10 min of centrifugation at 1,800 × g, the cells were harvested from the Percoll interface, suspended in 8 mL of L15 medium, and then centrifuged again for 5 min at 1,800 × g. The supernatant was removed, and the cell pellet was resuspended in 70 μL of DMEM containing 10% heat-inactivated foetal bovine serum (Sigma), penicillin G (100 U/mL), and streptomycin (100 μg/mL). The cells were plating onto laminin-coated coverslips and incubated at 37 °C. After 4 h of incubation, 1.5 mL of DMEM was added, and the cells were incubated overnight at 37 °C.

### Fluorometric Ca^2+^ imaging

Transfected HEK293 cells or cultured mouse DRG neurons on coverslips were loaded for 30–40 min with 5 μM fura-2 acetoxymethyl ester (Fura-2 AM; Dojindo Laboratories, Kumamoto, Japan) in Krebs-Ringer buffer containing (in mM) 140 NaCl, 5 KCl, 1 MgCl_2_, 2 CaCl_2_, 10 glucose, and 10 HEPES along with 0.01% Cremophor EL (Sigma-Aldrich). The fluorescence images of the cells were recorded and analysed using a time-lapse image analysis system (HC Image; Hamamatsu Photonics, Shizuoka, Japan) equipped with a digital camera (ORCA-Flash 4.0, Hamamatsu Photonic) and an inverted microscope (ECLIPSE Ti-E; Nikon, Tokyo, Japan) according to the manufacturers’ instructions. The fluorescence images were captured every 15 s using alternating excitation wavelengths of 340 and 380 nm, and an emission wavelength of 510 nm. The ratio of the fluorescence intensity obtained by the excitation/emission of 340 nm/510 nm (F_340_) to the fluorescence intensity obtained by the excitation/emission of 380 nm/510 nm (F_380_), namely F_340_/F_380_, was calculated to quantify [Ca^2+^]_i_. The cells with an F_340_/F_380_ ratio over 1.5 at baseline were excluded. The device was calibrated according to the manufacture’s protocol. In HEK293 cells treated with 4 μM ionomycin (Wako), the maximal F_340_/F_380_ ratio was 2.05 ± 0.001 in the presence of 2 mM Ca^2+^, and was saturated by more than 2 mM Ca^2+^. Once the calibrations were made, the device settings were fixed, and then measurements were made.

For statistical analyses, the change in the F_340_/F_380_ ratio (ΔRatio) was calculated from the baseline, which was defined as the average F_340_/F_380_ ratio for 3 min immediately before H_2_O_2_ application. In the experiments using HEK293 cells, the maximal ΔRatios during the 5 min H_2_O_2_ application period were calculated. In the experiments using mouse DRG neurons, H_2_O_2_-sensitive cells were defined as those having a maximal ΔRatio of more than 0.1 during the 5 min H_2_O_2_ application period. Statistical analyses were performed for the percentage of H_2_O_2_-sensitive cells within the 50 mM KCl-positive neurons. AITC and capsaicin were used to validate the expression of TRPA1 and TRPV1, respectively. To determine whether HEK293 cells or DRG neurons were alive, 3 μM ionomycin or 50 mM KCl was applied, respectively.

### Treatment with hypoxia and reoxygenation

The *P*O_2_ in the chamber was controlled with a Digital Gas Mixer GM-8000 (Tokai Hit, Shizuoka, Japan) under a Stage Top Incubator INUBSF-ZILCS (Tokai Hit, Shizuoka, Japan). The *P*O_2_ in Krebs-Ringer buffer was recorded using a Licox-JBOX (Medical Agent, Kyoto, Japan). The *P*O_2_ was reduced at a rate of approximately 2.7 mmHg/min to 100, 90 or 80 mmHg. The cells were exposed to the hypoxic condition for 30 min, and then the *P*O_2_ was rapidly returned to normoxia (160 mmHg) using the Digital Gas Mixer and by adding a double volume of normoxic buffer containing H_2_O_2_.

### Statistical analysis

The data were analysed using GraphPad Prism 5 (GraphPad Software, Inc., La Jolla, CA) and are presented as the means ± S.E.M. Differences between two groups were compared using Student’s *t*-test. Data with more than two groups were compared using one-way or two-way analyses of variance (ANOVA), followed by the Bonferroni *post hoc* test. Time-course data were analysed using two-way ANOVA for repeated measures, followed by the Bonferroni *post hoc* test. In all cases, differences of *p *< 0.05 were considered statistically significant.

## Additional Information

**How to cite this article**: So, K. *et al.* Hypoxia-induced sensitisation of TRPA1 in painful dysesthesia evoked by transient hindlimb ischemia/reperfusion in mice. *Sci. Rep.* **6**, 23261; doi: 10.1038/srep23261 (2016).

## Supplementary Material

Supplementary Information

Supplementary Movie
