# Supplementary material for: Hypoxia-induced sensitisation of TRPA1 in painful dysesthesia evoked by transient hindlimb ischemia/reperfusion in mice
Source: Sci Rep. 2016 Mar 17;6:23261. doi: 10.1038/srep23261 (PMC4794653; doi:10.1038/srep23261)
Supplement: Supplementary Information [file srep23261-s1.pdf]

Supplementary Information

Hypoxia-induced sensitisation of TRPA1 in painful dysesthesia evoked by transient hindlimb ischemia/reperfusion in mice

Kanako So[1], Yuna Tei[2], Meng Zhao[1], Takahito Miyake[1], Haruka Hiyama[1], Hisashi Shirakawa[1], Satoshi Imai[2], Yasuo Mori[3], Takayuki Nakagawa[2,*], Kazuo Matsubara[2], Shuji Kaneko[1]

[1]Department of Molecular Pharmacology, Graduate School of Pharmaceutical Sciences, Kyoto University, 46-29 Yoshida-Shimoadachi-cho, Sakyo-ku, Kyoto 606-8501, Japan

[2]Department of Clinical Pharmacology and Therapeutics, Kyoto University Hospital, 54 Shogoin-Kawahara-cho, Sakyo-ku, Kyoto 606-8507, Japan

[3]Department of Synthetic Chemistry and Biological Chemistry, Graduate School of Enginnering, Kyoto University, Katsura Campus, Nishikyo-ku, Kyoto 615-8510, Japan

***Corresponding author:** Takayuki Nakagawa

Department of Clinical Pharmacology and Therapeutics, Kyoto University Hospital

54 Shogoin-Kawahara-cho, Sakyo-ku, Kyoto 606-8507, Japan

Tel/ Fax: +81-75-751-4560

E-mail: tknakaga@kuhp.kyoto-u.ac.jp

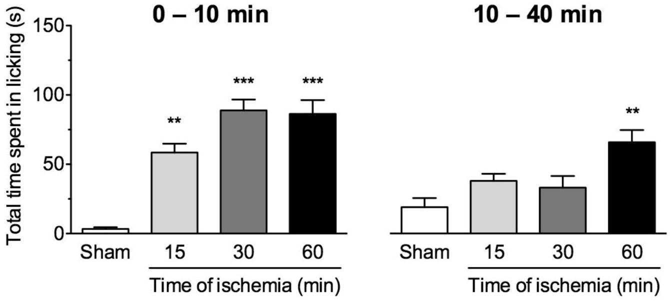

Supplementary Fig. S1. Longer hindlimb ischemia followed by reperfusion evokes biphasic spontaneous licking. Mice were exposed to hindlimb ischemia for 60 min followed by relief from the ischemia, as shown in Figure 3b. The time spent licking in the early phase (left panel; 0 – 10 min) and delayed phases (right panel; 10 – 40 min) were calculated. The early phase of liking was significantly increased ($F_{3,29}$ = 18.2, $p < 0.001$) by 15-, 30- and 60-min hindlimb ischemia, while the delayed phase was significantly increased ($F_{3,29}$ = 6.23, $p < 0.01$) only by 60-min, but not 15- and 30-min, hindlimb ischemia. n = 4–15, **$P < 0.01$, ***$P < 0.001$ vs sham treatment.

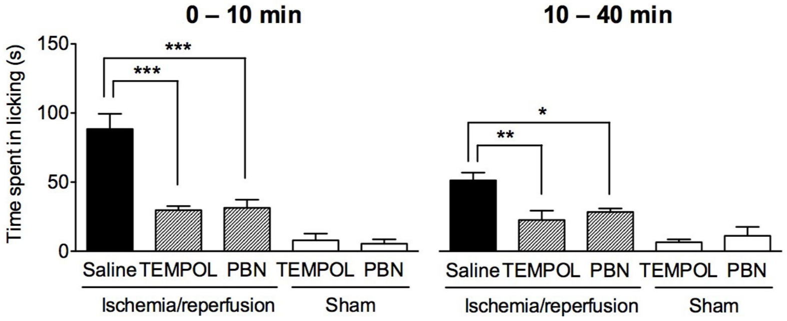

Supplementary Fig. S2. Effects of ROS scavengers on the early and delayed phases of hindlimb ischemia/reperfusion-evoked spontaneous licking. TEMPOL (250 mg/kg), PBN (100 mg/kg), or vehicle was administered i.p. 15 min before reperfusion following 60-min hindlimb ischemia or sham treatment, as shown in Fig. 4b. The time spent licking in the early (left panel; 0 – 10 min) and delayed phases (right panel; 10 – 40 min) were calculated. Both the early ($F_{2,15}$ = 20.4, p < 0.001) and delayed phases ($F_{2,15}$ = 8.13, p < 0.01) of liking were significantly attenuated by TEMPOL or PBN. n = 6, *P < 0.05, **P < 0.01, ***P < 0.001.

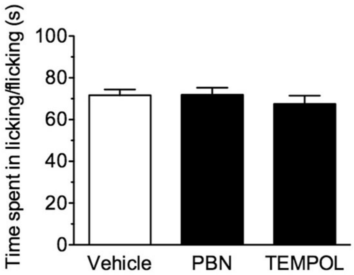

Supplementary Fig. S3. Effects of ROS scavengers on the TRPA1 agonist-evoked nocifensive behaviours. A TRPA1 agonist, AITC-evoked nocifensive behaviour was measured, as previously described[29] with slight modifications. Mice were individually habituated to the experimental conditions in an acrylic observation cylinder for approximately 1 h before AITC injection. TEMPOL (250 mg/kg), PBN (100 mg/kg), or vehicle was administered intraperitoneally 15 min before the test. Then, AITC (0.1%, 20 μL/paw) diluted in corn oil was subcutaneously injected into the plantar surface of the right hindpaw, and the time spent in nocifensive behaviours, such as licking and flicking of the injected hindpaw, was measured for 5 min. n = 6. There was no difference in the AITC-evoked nocifensive behaviours among groups ($F_{2,15} = 0.53$, $p = 0.60$).

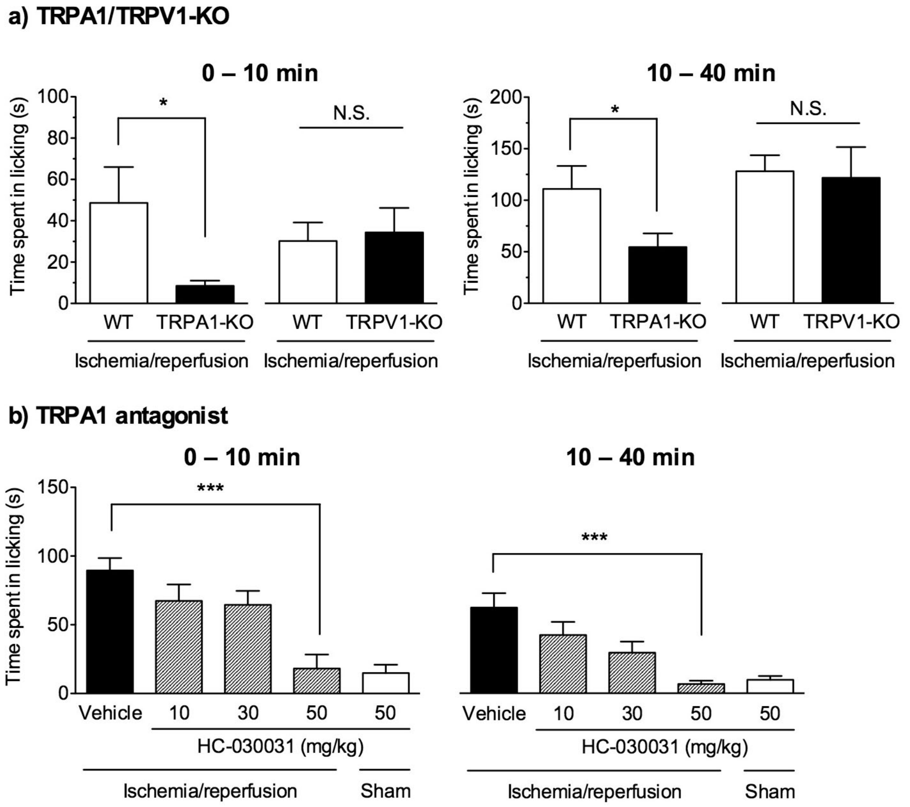

Supplementary Fig. S4. Effects of TRPA1 deficiency and a TRPA1 antagonist on the early and delayed phases of hindlimb ischemia/reperfusion-evoked spontaneous licking. (a) Wild-type (WT), TRPA1-knockout (KO) or TRPV1-KO mice were subjected to hindlimb ischemia/reperfusion, as shown in Fig. 5c. The time spent licking in the early phase (left panel; 0 – 10 min) and delayed phases (right panel; 10 – 40 min) were calculated. Both the early and delayed phases of liking were significantly attenuated in TRPA1-KO mice compared with that in WT mice, whereas they remained unchanged in TRPV1-KO mice. $n = 5–9$. $*P < 0.05$. N.S. = not significant. (b) The TRPA1 antagonist HC-030031 (10, 30, and 50 mg/kg) or vehicle was administered i.p. immediately before initiating ischemia or in sham-treated mice, as shown in Fig.5d. The time spent licking in the

early (left panel; 0 – 10 min) and delayed phases (right panel; 10 – 40 min) were calculated. Both the early phase ($F_{3,21}$ = 8.48, $p < 0.001$) and delayed phases ($F_{3,21}$ = 6.85, $p < 0.01$) of liking were significantly attenuated by HC-030031. n = 5–7, **P < 0.01, ***P < 0.001.

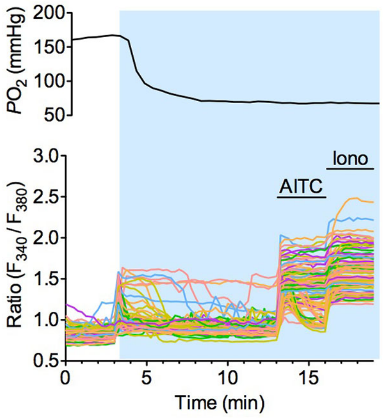

Supplementary Fig. S5. Abrupt hypoxia induces robust TRPA1 activation.

In HEK293 cells expressing human TRPA1, the O_2 concentration in the Krebs-Ringer buffer was abruptly decreased to a partial pressure (PO_2) of 80 mmHg (10.5% O_2), and the PO_2 in the buffer and the intracellular Ca^{2+} concentration ($[Ca^{2+}]_i$) were measured. Representative images of the PO_2 (upper trace) and $[Ca^{2+}]_i$ changes (F_{340}/F_{380} ratio; lower traces) recorded in individual cells are shown. AITC, allyl isothiocyanate, 100 μM; Iono, ionomycin, 3 μM.

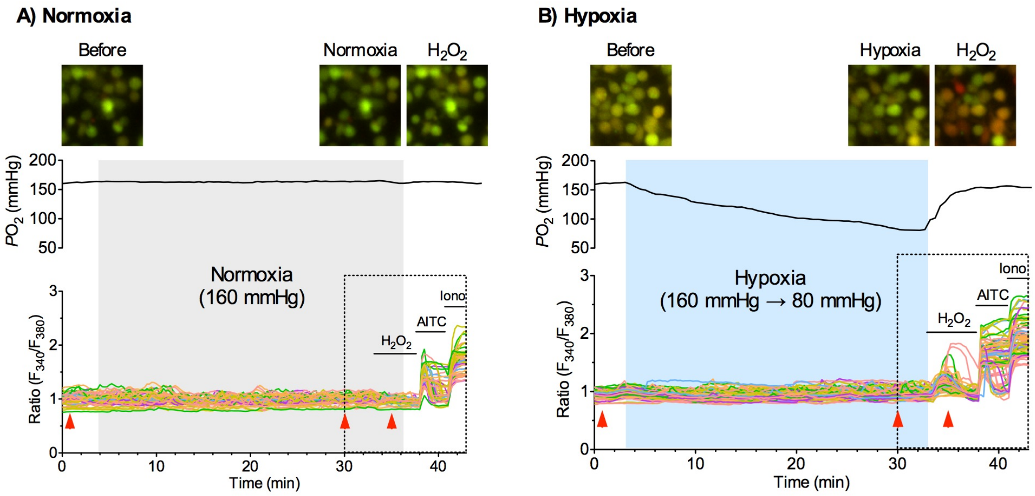

Supplementary Fig. S6. Gradual hypoxia elicits little TRPA1 activation but induces TRPA1 sensitization to H_2O_2. In HEK293 cells expressing human TRPA1, the O_2 concentration in Krebs-Ringer buffer was maintained at an atmospheric partial pressure (PO_2) of 160 mmHg (a; normoxia) or gradually decreased to a hypoxic PO_2 of 80 mmHg for 30 min (b; hypoxia). The O_2 concentration was then quickly returned to the atmospheric PO_2 of 160 mmHg, while H_2O_2 (10 μM) dissolved in a double volume of normoxic buffer was concomitantly applied to the cells. Allyl isothiocyanate (AITC; 100 μM) and ionomycin (Iono, 3 μM) were applied to validate the expression of TRPA1 and to show that the cells were alive, respectively. During all treatments, the PO_2 (mmHg) in Krebs-Ringer buffer and the intracellular Ca^{2+} concentration $[Ca^{2+}]_i$ (F_{340}/F_{380} ratio) were recorded. Representative images of fluorescence (upper panels), F_{340}/F_{380} ratios (lower panels) recorded in individual cells, and PO_2 of the buffer (middle panels) are shown. Red triangles indicate when the fluorescence images shown in the upper panels were captured.

Supplementary Video 1. Spontaneous licking following transient hindlimb ischemia/reperfusion. The mouse receiving reperfusion following a transient hindlimb ischemia for 60 min was placed in the centre of the observation cylinders, and the spontaneous behaviours were videotaped. The video shows spontaneous licking to the ipsilateral hindpaw at the peak time (4 – 5 min after reperfusion).
